# Serum *N*‐Glycome analysis reveals pancreatic cancer disease signatures

**DOI:** 10.1002/cam4.3439

**Published:** 2020-09-08

**Authors:** Gerda C. M. Vreeker, Randa G. Hanna‐Sawires, Yassene Mohammed, Marco R. Bladergroen, Simone Nicolardi, Viktoria Dotz, Jan Nouta, Bert A. Bonsing, Wilma E. Mesker, Yuri E. M. van der Burgt, Manfred Wuhrer, Rob A. E. M. Tollenaar

**Affiliations:** ^1^ Department of Surgery Leiden University Medical Center Leiden The Netherlands; ^2^ Center for Proteomics and Metabolomics Leiden University Medical Center Leiden The Netherlands

**Keywords:** cancer biomarker analysis, mass spectrometry‐based *N*‐glycan profiling, *N*‐glycome analysis, pancreatic cancer, serum test

## Abstract

**Background &Aims:**

Pancreatic ductal adenocarcinoma (PDAC) is an aggressive cancer type with loco‐regional spread that makes the tumor surgically unresectable. Novel diagnostic tools are needed to improve detection of PDAC and increase patient survival. In this study we explore serum protein *N*‐glycan profiles from PDAC patients with regard to their applicability to serve as a disease biomarker panel.

**Methods:**

Total serum *N*‐glycome analysis was applied to a discovery set (86 PDAC cases/84 controls) followed by independent validation (26 cases/26 controls) using in‐house collected serum specimens. Protein *N*‐glycan profiles were obtained using ultrahigh resolution mass spectrometry and included linkage‐specific sialic acid information. *N*‐glycans were relatively quantified and case‐control classification performance was evaluated based on glycosylation traits such as branching, fucosylation, and sialylation.

**Results:**

In PDAC patients a higher level of branching (OR 6.19, *P*‐value 9.21 × 10^−11^) and (antenna)fucosylation (OR 13.27, *P*‐value 2.31 × 10^−9^) of *N*‐glycans was found. Furthermore, the ratio of α2,6‐ vs α2,3‐linked sialylation was higher in patients compared to healthy controls. A classification model built with three glycosylation traits was used for discovery (AUC 0.88) and independent validation (AUC 0.81), with sensitivity and specificity values of 0.85 and 0.71 for the discovery set and 0.75 and 0.72 for the validation set.

**Conclusion:**

Serum *N*‐glycome analysis revealed glycosylation differences that allow classification of PDAC patients from healthy controls. It was demonstrated that glycosylation traits rather than single *N*‐glycan structures obtained in this clinical glycomics study can serve as a basis for further development of a blood‐based diagnostic test.

## Graphical abstract

Serum protein glycosylation differences between pancreatic cancer patients and healthy individuals are discussed with regard to their potential to function as a diagnostic signature. Results were validated in an independent cohort and it was concluded that the serum N‐glycome analysis can serve as a basis for the development of a blood‐based diagnostic test for the detection of pancreatic cancer.

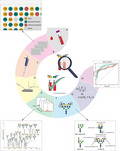

## 1 INTRODUCTION

Pancreatic ductal adenocarcinoma (PDAC) is the most common type of pancreatic cancer with an increasing incidence in western countries.^1^ Diagnosis of PDAC implies an unfavorable prognosis with five‐year survival as low as 5%‐8%, since the disease is characterized by aggressive local and early metastatic spread. Upon initial diagnosis more than 80% of the tumors is at an advanced stage that does not allow curative resection.^2^ Intensive treatment schedules with chemotherapy and/or surgery are associated with complications, side effects and impaired quality of life, while overall survival remains poor.^3^ A recent study reported that 10% of the PDAC patients carry a *BRCA* gene mutation which could provide an opportunity to apply screening and use targeted treatment to improve outcome.^4^ It is furthermore noted that the number of PDAC deaths is not far from that of, for example, breast cancer, and a future screening for PDAC may be warranted.^5^, ^6^, ^7^, ^8^, ^9^ However, screening programs based on current detection methods that comprise imaging techniques and/or fine‐needle‐aspirations, are not feasible.^10^, ^11^ Moreover, chronic and autoimmune pancreatitis (CP) can mimic PDAC and consequently cause a 5%‐10% misclassification.^12^ For these reasons, new blood‐based biomarker tests are pursued that offer a more cost‐effective way to detect the disease.^13^ This urgent need for additional biomarkers to facilitate clinical decision‐making is widely acknowledged, since the only marker available is carbohydrate antigen (CA) 19‐9, which is primarily used for patient follow‐up (recurrent disease) and has limited value for the detection of PDAC.^14^

Mass spectrometry (MS)‐based biomarker studies have shown that posttranslational modifications (PTMs) hold potential as an “add‐on” to the protein marker or as biomarkers themselves.^13^, ^15^, ^16^ It is well known that a single gene does not transcribe and translate into a single protein but rather in a plethora of proteoforms and that proteome characterization should include the analysis of PTMs.^17^ In this context, the relevancy of protein glycosylation has been demonstrated in autoimmune diseases and cancer.^18^, ^19^, ^20^, ^21^ In‐depth glycobiology studies have furthermore revealed the importance of protein glycosylation with regard to folding, trafficking, cell adhesion, recognition processes, and immune response.^22^, ^23^ Notably, the previously mentioned marker CA19‐9 is a glycan marker, based on a sialyl‐Lewis A (sLe^A^) epitope, which triggered interest in protein glycosylation related to pancreatic cancer. A few studies on *N*‐glycosylation profiles in pancreatic cancer have exemplified a biomarker potential, although sample sets were limited.^24^, ^25^, ^26^, ^27^, ^28^, ^29^, ^30^ Here, we use an automated protocol for the analysis of the total serum *N*‐glycome with sialic acid linkage differentiation and high resolution MS^31^ and aim for a PDAC disease signature in a discovery cohort with independent validation.

## 2 MATERIALS AND METHODS

### 2.1 Patients

Blood samples in the discovery cohort were obtained from 88 patients diagnosed with PDAC and collected prior to surgery. An equal number of specimens was collected from healthy volunteers, which were partners or accompanying persons of included patients. All samples from cases and controls originated from a Dutch population and were matched by sex and age and sample collection date (ie, freezer storage duration) in both the discovery and validation cohort.^32^ All patients in the discovery cohort were seen at the outpatient clinic of the Leiden University Medical Center between October 2002 and December 2008. For an independent validation cohort, blood specimens were collected between June 2016 and March 2018. All selected patients in the discovery and validation cohorts were candidates for curative surgery. However, not all patients underwent surgery due to preoperative metastases. PDAC diagnosis consisted of a combination of annual abdominal magnetic resonance imaging, magnetic resonance cholangiopancreatography and/or optionally endoscopic ultrasound. Furthermore, all surgical specimens were examined according to routine histological evaluation and the extent of the tumor spread was assessed by TNM classification.^33^, ^34^

Blood samples in the validation cohort were obtained from twenty patients diagnosed with PDAC, two patients with duodenal and papillary carcinoma, two patients with neuroendocrine tumors and three patients with IPMN. A total of twenty‐seven healthy controls were randomly selected from the LUMC Biobank. Cases and controls were matched by sex and age and sample collection date (ie, freezer storage duration) in both the discovery and validation cohort.

This study was approved by the Medical Ethical Committee of the LUMC (protocol number P03‐147). All patients and healthy volunteers provided written informed consent prior to blood collection.

### 2.2 Serum sample collection and plate design

Blood specimens from both the discovery and validation cohorts were collected and processed according to a standardized protocol.^35^ Briefly, all blood samples were drawn by antecubital venipuncture. Approximately 8 mL of venous blood was collected in a 10 mL BD vacutainer SST II advance and centrifuged for 10 minutes at 1000*g*. Processing of blood specimens took place within 4 hours after blood collection. After the centrifugation step serum samples were distributed into sterile, 500‐μL barcode‐labeled aliquots and stored at −80°C until further analysis. Before measurements (ie, serum *N*‐glycome analysis) took place, each sample was aliquoted into 60 μL tubes.^35^ One aliquot of each sample was then relocated into a 96‐well plate format according to a plate design, thus keeping cases and their age‐ and sex‐matched controls on the same plate. Additionally, for technical quality control (QC) of the spectra, each plate contained a minimum of six in‐house standards and two blanks.

### 2.3 Serum sample preparation and mass spectrometry analysis of glycans

*N*‐glycans were enzymatically released from serum glycoproteins, chemically derivatized, purified, MS‐analyzed, identified and quantified. Briefly, 6 μL of serum was used according to a previously reported protocol.^31^ The global release of *N*‐glycans was performed using the enzyme PNGase F (Roche Diagnostics, Mannheim, Germany). All following steps were carried out in a standardized manner on a Hamilton liquid handling platform. In a first step, all sialic acid residues at the nonreducing ends of the complex glycan structures were derivatized into stable end‐products allowing the differentiation between α2,3‐ and α2,6‐linked sialic acids by the introduced mass difference. Next, the glycans were purified using in‐house developed cotton‐based hydrophilic interaction liquid chromatography (HILIC) micro‐tips. The purified glycans were eluted and premixed with sDHB matrix (5 mg/mL in 99% ACN with 1 mmol/L NaOH). The mixture was spotted onto a MALDI target plate (800/384 MTP AnchorChip, Bruker Daltonics, Bremen, Germany) and spots were allowed to dry. Measurements were performed on a Bruker 15T solariX XR Fourier transform ion cyclotron resonance (FTICR)MS. The system was controlled by ftms Control version 2.1.0 and spectra in an *m/z*‐range from 1011.86 to 5000.00 were recorded with 1 mmol/L data points (ie, transient length of 2.307 seconds). DataAnalysis Software 4.2 (Bruker Daltonics) was used for the visualization and data analysis of all MALDI‐FTICR spectra. Sample preparation and subsequent glycan measurements were identical for all samples in both cohorts, however the validation cohort was processed five months after the discovery cohort.

### 2.4 Data processing and statistics

Serum *N*‐glycan profiles were obtained from all 88 cases and 88 controls in the discovery cohort, of which 86 case‐profiles and 84 control‐profiles passed the quality criteria.^31^ These profiles are further referred to as the *discovery set*. In the validation cohort, consisting of 27 cases and 27 controls, 26 case‐profiles and 26 control‐profiles passed. These profiles are further referred to as the *validation set*. For both the discovery and validation set, the same analyte list with 84 glycan compositions(Table S1) which passed the quality criteria^31^ was used for data extraction with MassyTools version 0.1.8.1.^31^ To study general glycosylation features, such as fucosylation, branching, sialylation and bisection, derived traits were calculated to combine the effects of glycans with similar structures (Table S2).

To evaluate the potential of total serum *N*‐glycome analysis in differentiating PDAC patients from controls, logistic regression was performed for each glycoform individually as well as for each derived trait (Tables S3 and S4), using R version 3.3.2 (R Foundation for Statistical Computing, Vienna, Austria; Released 31 October 2016) and RStudio, version 1.0.136 (RStudio, Boston, MA; Released 21 December2016).^36^ The odds ratios (ORs) were calculated with their 95% confidence intervals (CIs) assuming a Student's *t*‐distribution and are referring to an increase of 1 SD in the tested traits. A fixed‐effects model was used to combine the data of the discovery and validation set in a meta‐analysis. Multiple testing correction (Bonferroni) was performed on the meta‐analyzed data. In order to evaluate potential trait differences between the various cancer stages, stages Ia, Ib and IIa were merged into one sub‐group, IIb was considered as a separate sub‐group, and stages III and IV were also merged into one subgroup. For plotting purposes, the center line is median, box limits are upper and lower quartiles, and whiskers give the maximum and minimum values excluding any outliers. All points are individual measurements and outliers are the individual measurements larger than quartile 3 + 1.5× IQR or smaller than quartile 1 ‐ 1.5× IQR (IQR = interquartile range). For all glycan comparisons between case‐control subjects the significance level is stated in each corresponding plot after adjusting the *P*‐value of Student's *t*‐test using B‐H method.

Receiver operating characteristic (ROC) analysis was performed by selecting derived traits representing the different glycosylation features that showed the strongest effect sizes (antennarity, fucosylation, sialylation) in the meta‐analysis. Initially, five derived traits were used for the model, namely CA2 (diantennary species of complex glycans in spectrum), CA4 (tetraantennary species of complex glycans in spectrum), A3FE (α2,6‐sialylation of fucosylated triantennary glycans), A3F0L (α2,3‐sialylation of nonfucosylated triantennary glycans) and CFa (antenna‐fucosylation of complex glycans). Multiple combinations of these traits were then evaluated with regard to classification of diagnosis, resulting in a final model based on a combination of CA4, A3F0L and CFa. The model was trained using a randomly selected 75% of the discovery set and evaluated for its prediction value on the remaining 25% to prevent overfitting. More importantly, the prediction was replicated on the validation set. The power of the classification (area under the curve) was evaluated ten times with each time a new random selection of 75% of the discovery set, resulting in a mean power that was more robust than a single classification.

## 3 RESULTS

**FIGURE 1:**
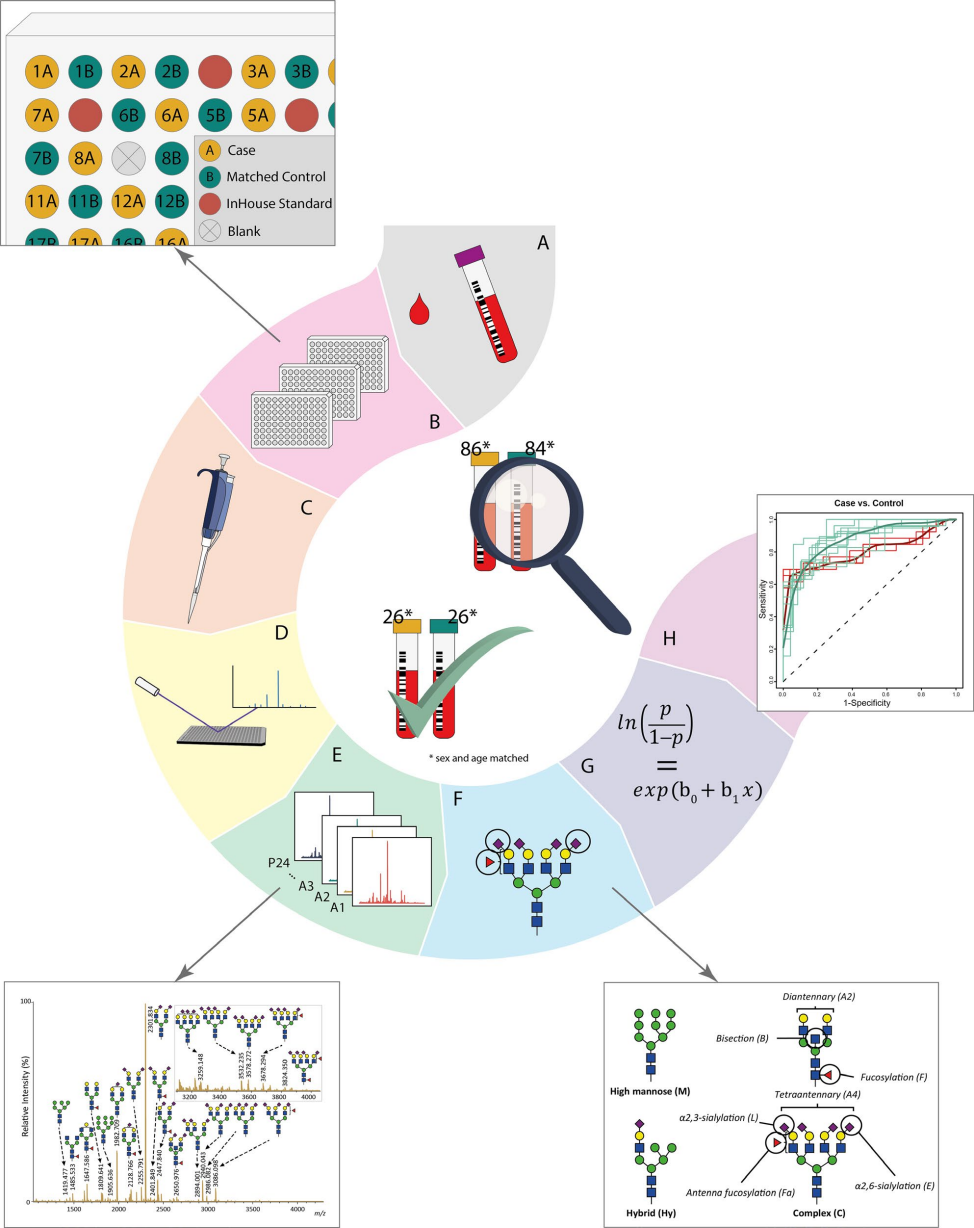
Workflow of *N*‐glycosylation analysis of discovery and validation pancreatic cancer case‐control cohorts for classification analysis. A, Collection of serum samples from pancreatic cancer patients and healthy controls. B, Random distribution of age‐and sex‐matched case‐control pairs, in‐house standards and blanks. C, Automated sample preparation including enzymatic glycan release, derivatization and purification. D, MALDI‐FTICR‐MS analysis of *N*‐glycome. E, MS‐spectrum preprocessing, annotation and quality control. F, Derived trait calculation for the analysis of glycosylation features. G, Logistic regression analysis of both cohorts, followed by meta‐analysis of the data. H, ROC analysis to test glycosylation traits for their classification power

The serum *N*‐glycomes of PDAC patients and matched controls in a discovery and independent validation set (Table 1) were analyzed by mass spectrometry. Derived traits were calculated for structural features shared by multiple glycans, such as the level of antennarity (in the following abbreviated as CA), α2,3‐linked sialylation (L), α2,6‐linked sialylation (E), fucosylation (F) and bisection (B) (Figure 1). Data of consistent quality were obtained as assessed from 19 in‐house standards that were included in the TSNG measurements. It is furthermore noted that the MS‐based glycan profiles provide relative quantitative data that do not explain whether differences are caused by different serum protein concentrations or to which extent protein‐specific glycosylation differences contribute. The data revealed age‐ and sex‐associations of the glycomic signatures (Figure S1) in accordance with literature^37^ supporting the validity of the data. Logistic regression analysis was performed both at the single *N*‐glycan level and the derived traits (Figure 1) revealing a total of 23 glycosylation features that where consistently found to differ between patients and controls as demonstrated by our meta‐analysis (Table 2).

**TABLE 1:** Patient characteristics

	Discovery Set		Validation Set	
	Cases	Controls	Cases	Controls
	(n = 86)	(n = 84)	(n = 26)	(n = 26)
Female sex, n (%)	47 (54.7)	45 (53.4)	11 (42.3)	11 (42.3)
Age in years, mean (SD)	64.6 (11.1)	63.2 (10.0)	66.3 (10.5)	66.7 (5.4)
Diagnosis, n				
PDAC	86	n/a	20	n/a
Other	n/a	n/a	6	n/a
Stage, n				
Ia	6	n/a	0	n/a
Ib	8	n/a	1	n/a
IIa	10	n/a	3	n/a
IIb	41	n/a	7	n/a
III	3	n/a	1	n/a
IV	18	n/a	8	n/a

Abbreviations: n, number of individuals; n/a, not applicable; SD, standard deviation.

**TABLE 2:** Replicated meta‐analyzed associations of serum *N*‐glycans with pancreatic cancer

Derived traits	Description of derived traits	Meta *P*‐values	Odds ratio	Confidence interval
		Cases/Controls	Cases/Controls	Cases/Controls
Glycan type				
CA2	Diantennary species of complex glycans in spectrum	1.05E‐08	0.35	(0.25‐0.50)
CA4	Tetraantennary species of complex glycans in spectrum	9.21E‐11	6.19	(3.57‐10.75)
CFa	Antenna‐fucosylation of complex glycans	2.31E‐09	13.27	(5.68‐30.98)
CB0	Nonbisected species of complex glycans in spectrum	5.12E‐08	0.39	(0.27‐0.54)
Fucosylation (F)				
A3F	Fucosylation of triantennary glycans	2.07E‐07	2.34	(1.70‐3.23)
A4F	Fucosylation of tetraantennary glycans	5.00E‐06	2.04	(1.50‐2.78)
A3Fa	Antenna‐fucosylation of triantennary glycans	1.12E‐08	5.35	(3.01‐9.52)
A4Fa	Antenna‐fucosylation of tetraantennary glycans	1.45E‐06	2.45	(1.70‐3.53)
A2LF	Fucosylation of α2,3‐sialylated diantennary glycans	3.85E‐08	2.67	(1.88‐3.78)
A3LF	Fucosylation of α2,3‐sialylated triantennary glycans	9.32E‐09	2.68	(1.91‐3.75)
A4LF	Fucosylation of α2,3‐sialylated tetraantennary glycans	1.70E‐06	2.13	(1.56‐2.91)
A4EF	Fucosylation of α2,6‐sialylated tetraantennary glycans	6.06E‐06	2.03	(1.49‐2.75)
Sialylation (S)				
A4F0S	Sialylation of nonfucosylated tetraantennary glycans	3.07E‐06	0.48	(0.35‐0.65)
A3FS	Sialylation of fucosylated triantennary glycans	7.41E‐05	1.95	(1.40‐2.71)
A4FS	Sialylation of fucosylated tetraantennary glycans	2.85E‐06	2.1	(1.54‐2.87)
α2,3‐Linked sialylation (L)				
A2F0L	α2,3‐sialylation of nonfucosylated diantennary glycans	5.74E‐07	0.38	(0.26‐0.55)
A3F0L	α2,3‐sialylation of nonfucosylated triantennary glycans	8.34E‐09	0.34	(0.23‐0.49)
A4F0L	α2,3‐sialylation of nonfucosylated tetraantennary glycans	4.50E‐07	0.44	(0.32‐0.61)
α2,6‐Linked sialylation (E)				
A3E	α2,6‐sialylation of triantennary glycans	3.22E‐07	2.41	(1.72‐3.38)
A2F0E	α2,6‐sialylation of nonfucosylated diantennary glycans	3.18E‐07	2.44	(1.73‐3.43)
A3F0E	α2,6‐sialylation of nonfucosylated triantennary glycans	2.62E‐09	3.5	(2.32‐5.30)
A3FE	α2,6‐sialylation of fucosylated triantennary glycans	1.20E‐09	3.99	(2.55‐6.24)
A4FE	α2,6‐sialylation of fucosylated tetraantennary glycans	2.02E‐08	2.63	(1.88‐3.69)

Note

Dark grsy and light gray shading indicate positive and negative associations, respectively, with the healthy controls being the reference. See Table S3 for the complete list of tests performed.

**FIGURE 2:**
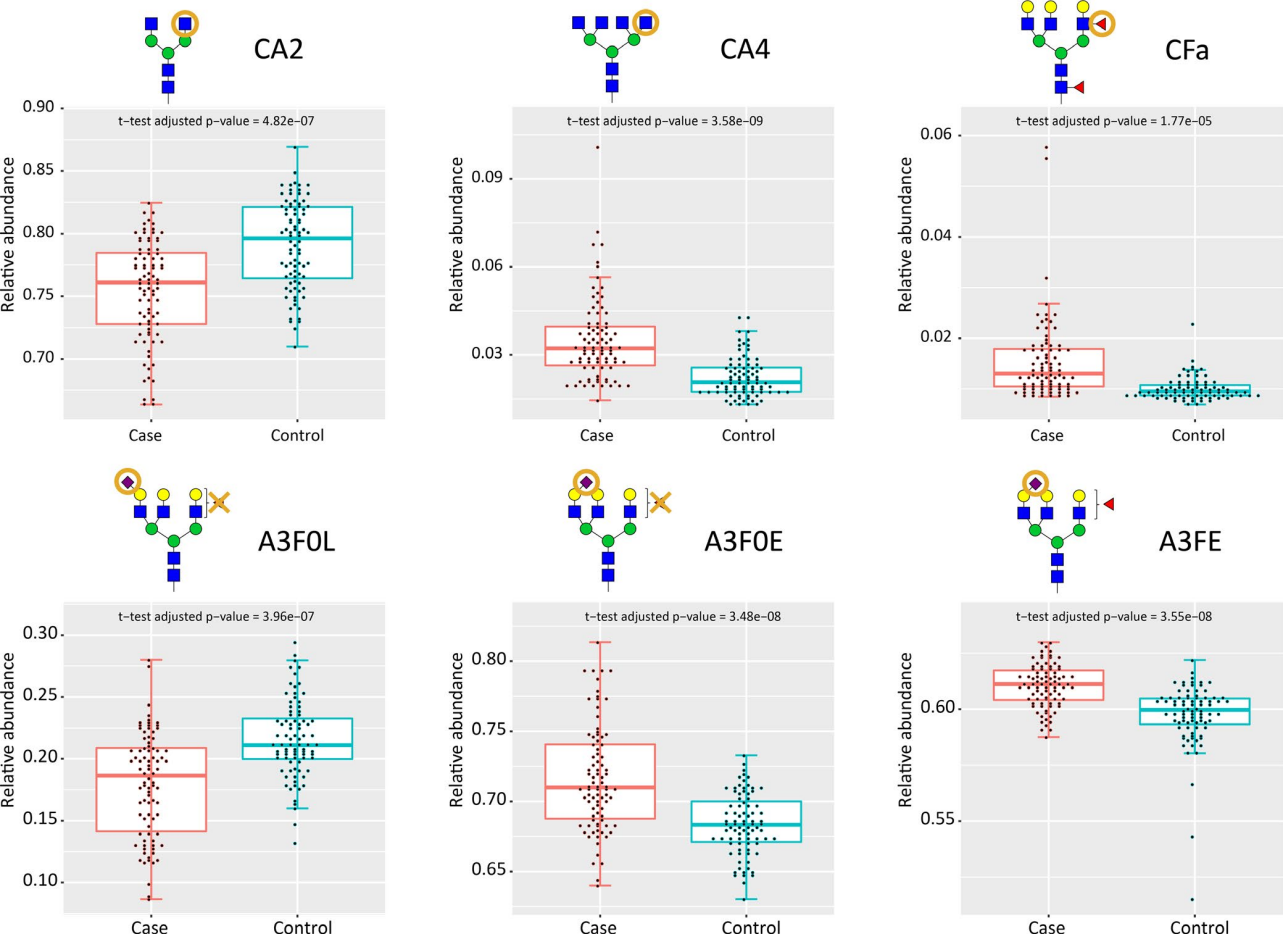
Main replicated associations between N‐glycan traits and pancreatic cancer, based on the data from the discovery cohort with corresponding Student's *t*‐test adjusted *P*‐values

A strong increase in the antennarity of the glycans was found: tetraantennary *N*‐glycans (CA4) were more abundant in PDAC profiles than in control samples (Table 2 and Figure 2), with a concomitant decrease in diantennary *N*‐glycans (CA2; Table 2 and Figure 2). Moreover, antenna‐fucosylation (CFa, difucosylation) was increased (CFa; Table 2 and Figure 2). Both mono‐ and difucosylation (F and Fa, respectively) were increased for tri‐ and tetraantennary glycans (A3F, A4F, A3Fa, A4Fa; Table 2).

Also, an increase in α2,6‐linked sialylation and a decrease in α2,3‐linked sialylation was observed (A2F0L, A3F0L, A4F0L; Table 2). Increased α2,6‐linked sialylation was observed in both fucosylated and non‐fucosylated di‐, tri‐ and tetraantennary glycans (eg, A3E, A3F0E, A3FE; Table 2).We further evaluated whether glycan derived traits were associated with cancer stages such as depicted in Table 1, but no differences were found between the various stages (details explained in the Methods section).

**FIGURE 3:**
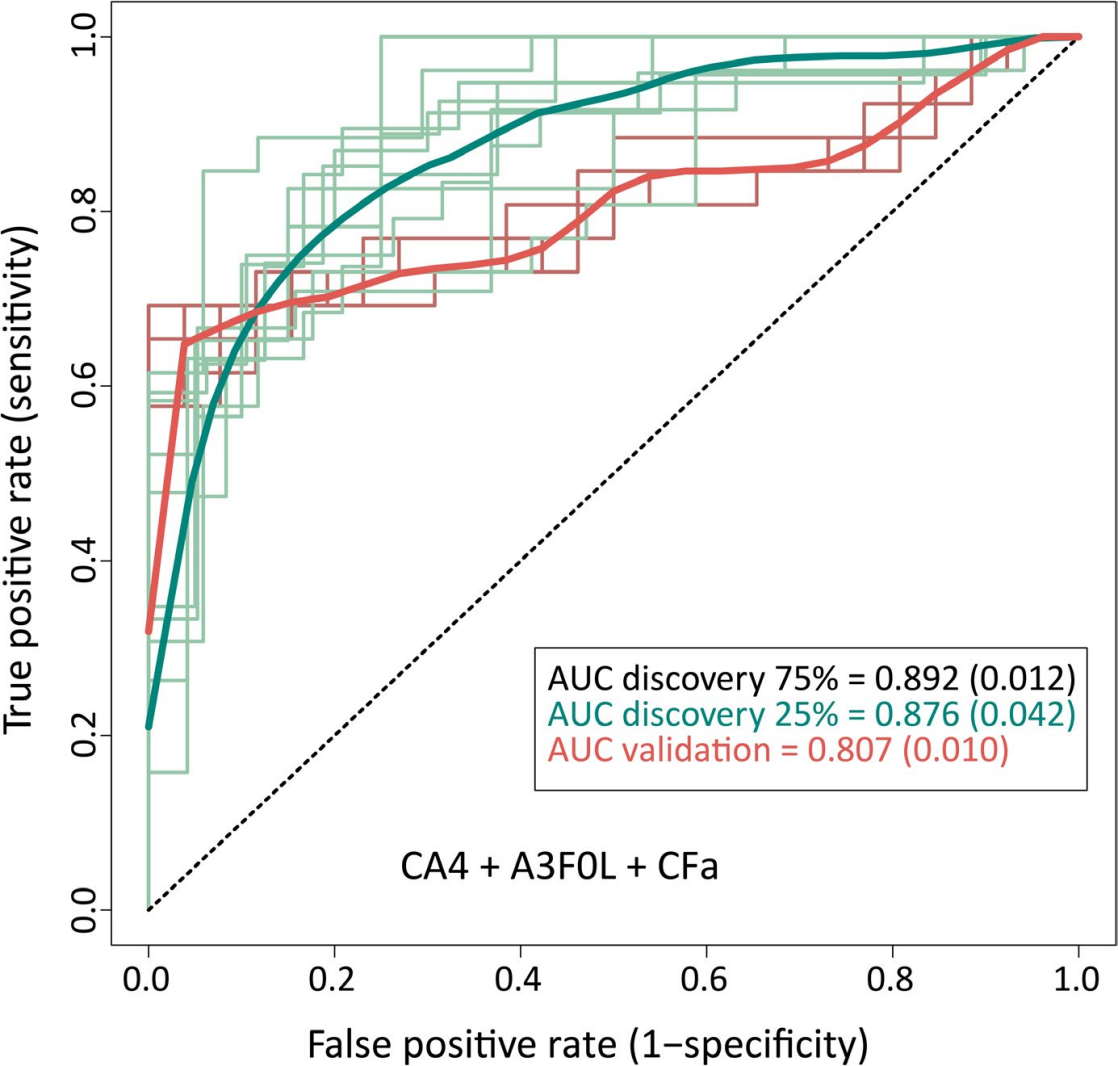
ROC analysis with a model based on CA4, A3F0L and CFa. The model was trained with a random selection of 75% of the spectra in the discovery cohort and applied to the remaining 25% of the cohort to test for its prediction value. Moreover, it was applied to an independent validation cohort to test for its classification power. This analysis was repeated ten times, to increase the robustness of AUCs. The means (and SDs) of 10 predictions are reported for the respective AUC

Finally, receiver operating characteristic (ROC) curves were calculated for selected glycan traits. The resulting ROC curve illustrates the power of differentiating PDAC from matched control samples (Figure 3). With an AUC of 0.88 the discriminative performance of the discovery set was good. At the optimal case probability score cut‐off, the sensitivity and specificity were 0.85 and 0.71, respectively. The signature was replicated in an independent validation cohort with a good AUC of 0.81, and with a sensitivity and specificity of 0.72 and 0.75, respectively.

## 4 DISCUSSION

The objective of this study was to explore the potential of serum protein *N*‐glycan profiles from PDAC patients to serve as a biomarker panel, aiming for the development of a blood‐based test for diagnosis of PDAC. Using our recently established analytical glycomics platform, 112 patient sera were analyzed and compared to 110 healthy control samples. The observed *N*‐glycosylation changes in the discovery cohort of PDAC patients were replicated in an independent validation cohort.

Major glycosylation differences were found between PDAC patients and controls for *N*‐glycan antennarity, fucosylation and sialylation. Notably, with regard to sialylation, our approach included an evaluation of α2,3‐ and α2,6‐linked sialic acids separately. We found twenty‐three glycosylation traits to be associated with PDAC in a meta‐analysis of the two sample sets.

PDAC patients showed higher α 2,6‐linked sialylation than controls. From tumor cell surface analysis it is known that an increase in overall α2,6‐linked sialic acids associates with cancer progression.^38^, ^39^ A possible explanation is that α 2,6‐linked sialic acids promote cancer cell survival since binding of proapoptotic galectins to cell surface glycans is blocked by these structures. In contrast, α 2,3‐linked sialic acids do not inhibit galectin binding.^38^, ^39^ With regard to protein *N*‐glycosylation, similar functions of α 2,6‐linked sialylation have been suggested, however the mechanisms in this case are not yet understood.^39^

This study also demonstrated elevated levels of tri‐ and tetraantennary *N*‐glycans and a concomitant decrease of diantennary *N*‐glycans in PDAC patients. Previously, similar observations in PDAC patients have been reported with regard to branching in total serum glycosylation profiles as well as in studies on specific glycoproteins, such as α‐1‐acid glycoprotein (AGP) and haptoglobin.^24^, ^25^, ^26^, ^40^ The first study reported elevated levels of tri‐ and tetraantennary glycans, however with a limited sample set of two pairs of cancer and normal samples only.^24^ The latter studies reported increased branching of AGP‐derived glycans with limited sample sets of 19 PDAC patients, six chronic pancreatitis patients and six controls, and increased branching in HPT and transferrin.^25^, ^26^ Increased tri‐ and tetraantennary glycans have furthermore been reported in association with progression of disease in sera and cell lines from PDAC patients.^24^, ^27^, ^28^ With regard to other cancer types, an increase in branching has been observed in brain and colorectal cancer.^21^, ^41^, ^42^, ^43^

Besides increased branching, an increased fucosylation of tri‐ and tetraantennary *N‐*glycans was found in PDAC patients. Increased fucosylation has been reported in various types of cancer such as hepatocellular carcinoma, oral and colorectal cancer.^21^, ^44^, ^45^ Also the previously mentioned glycoprotein studies on AGP and HPT reported increased fucosylation.^25^, ^26^ Interestingly, Akimoto and coworkers studied serum *N*‐glycan profiles of 79 patients with IPMN and found a potential marker for invasive IPMNs based on an increased expression of fucosylated complex‐type glycans. Unfortunately, the *N*‐glycan profiles in this study were not compared to those obtained from healthy control individuals.^40^

We found an increase of fucosylation of triantennary and tetraantennary glycans (A3F and A4F) in PDAC patients, specifically, in glycans containing α 2,3‐linked sialic acids (A2LF, A3LF and A4LF). The combination of α 2,3‐linked sialylation with fucosylation suggests the formation of sialyl‐Lewis X (sLe^x^) moieties. The increase of sLe^x^ expression has been reported with regard to pancreatic cancer.^26^, ^28^ In addition, increased sLe^x^ expression on AGP and HPT has been linked to various cancers (eg, pancreatic cancer, lung cancer, advanced ovarian cancer and prostate cancer)^18^, ^25^, ^46^, ^47^, ^48^, ^49^ and chronic inflammation (eg, rheumatoid arthritis and inflammatory bowel disease).^50^ As discussed in the Introduction, the marker CA19‐9 is based on a sialyl‐Lewis A (sLe^A^) epitope. Although this structure differs from sLe^x^ with regard to glycosidic linkages, both point toward the importance of sialylation.

Increased branching and sLe^x^ expression have been found in acute phase proteins which are released by the liver in the event of cancer, but also in case of infection, surgery and inflammatory conditions.^50^ The relation between inflammation and cancer has been discussed in a review, with the hypothesis that both glycosylation changes are a systemic side effect of inflammatory cytokines stimulating the liver under influence of the tumor.^51^ It has been demonstrated that the tumor microenvironment contains large amounts of these cytokines and that inflammatory pathways are involved in the development of tumors.^52^ The expression of these cytokines was also confirmed in studies on cell lines and tissues.^53^

The here applied glycomics workflow is specifically suited for a high‐throughput and relatively fast “cancer glycosylation profiling” of body fluids and cell or tissue material from a clinical cohort. This strategy does not provide detailed information on the protein origin of the potential glycan markers. This limitation is well known in the glycobiology community and can be tackled with in‐depth glycoproteomic analyses that come with their own challenges. Thus, although the analysis of total serum N‐glycosylation shows strong associations with PDAC, it is expected that analysis of specific glycoproteins might further improve accuracy.

The need for a screening test for pancreatic cancer is high, especially for patients with increased inherited risk. A screening test should meet specific requirements and should exhibit suitable sensitivity and specificity specifications.^29^ The current results are promising in terms of the discriminative performance for sensitivity and specificity, but translation into the clinic depends on the application. The screening of patients with a genetically increased risk for PDAC would be a first step since no testis currently available to support clinicians. For the general population the discriminative performance found in this study might be insufficient for application but could possibly be complementary to the CA 19‐9 test.^14^ Detection of PDAC at an earlier stage needs further investigation, since early detection is an important argument for population screening.^6^

The discriminating performance of case‐control ROC‐analysis was good, indicating a strong difference in *N*‐glycosylation profiles of PDAC patients and healthy controls. However, as indicated above, the *N‐*glycosylation shift we found in PDAC patients is not necessarily specific for pancreatic cancer. In this study, only PDAC patients and healthy volunteers were included, while in a clinical application other diseases might interfere with the determination of the PDAC cases. To address the specificity of the discriminating signals in this study, future research should compare PDAC signatures with those of benign diseases (eg, pancreatitis) as well as other types of cancers and inflammatory diseases.^30^

## 5 CONCLUSIONS

In this study, serum *N*‐glycome analysis with sialic acid isomer differentiation and ultrahigh resolution MS was performed to classify PDAC patients from healthy controls. Three major *N*‐glycosylation differences were observed and validated between cases and healthy controls, namely (antenna‐) fucosylation of complex glycans, branching of complex glycans and increased α 2,6‐linked sialylation compared to the α 2,3‐linked analogues. Combination of various *N*‐glycosylation traits resulted in classification performance that can function as a target for follow‐up glycomics research aiming for development of a blood‐based clinical test. In future research the specificity of the observed changes needs to be addressed by including samples from benign pancreatic diseases including inflammation and preferably other cancer types. In addition, longitudinal analysis is warranted to determine the potential for early detection based on the here reported serum *N*‐glycan disease signatures.

## CONFLICT OF INTEREST

The authors declare that there is no conflict of interest.

## AUTHOR CONTRIBUTIONS

RT, WM and MW conceptualized the study and acquired funding. RHS collected the sample cohorts. GV, MB, SN, and JN performed the experiments. GV performed data curation and validation. GV, YM and VD performed the formal analysis. GV, YM and MB visualized the data. GV, RHS and YB wrote the original draft of the manuscript and all authors were involved in reviewing and editing of the manuscript. MW, RT, WM, YB, and BB were involved in the supervision.

## Supporting information

Fig S1

Table S1‐S4

## Data Availability

All data generated during in this study are stored in‐house and can be shared upon request. The well‐established repositories for mass spectrometry data, such as MassIVE (massive.ucsd.edu) and PRIDE (https://www.ebi.ac.uk/pride/) do not support glycomics data yet. Since PRIDE is the most mature of all mass spectrometry repositories, that allows systematic sample annotation and metadata submission, we foresee future inclusion of glycomics data. This will make our data findable and reusable (as in FAIR).
