# Supplementary material for: Serum N‐Glycome analysis reveals pancreatic cancer disease signatures
Source: Cancer Med. 2020 Sep 8;9(22):8519–29. doi: 10.1002/cam4.3439 (PMC7666731; doi:10.1002/cam4.3439)
Supplement: Supplementary file 1 — Fig S1 [file CAM4-9-8519-s001.pptx]

## Slide 1
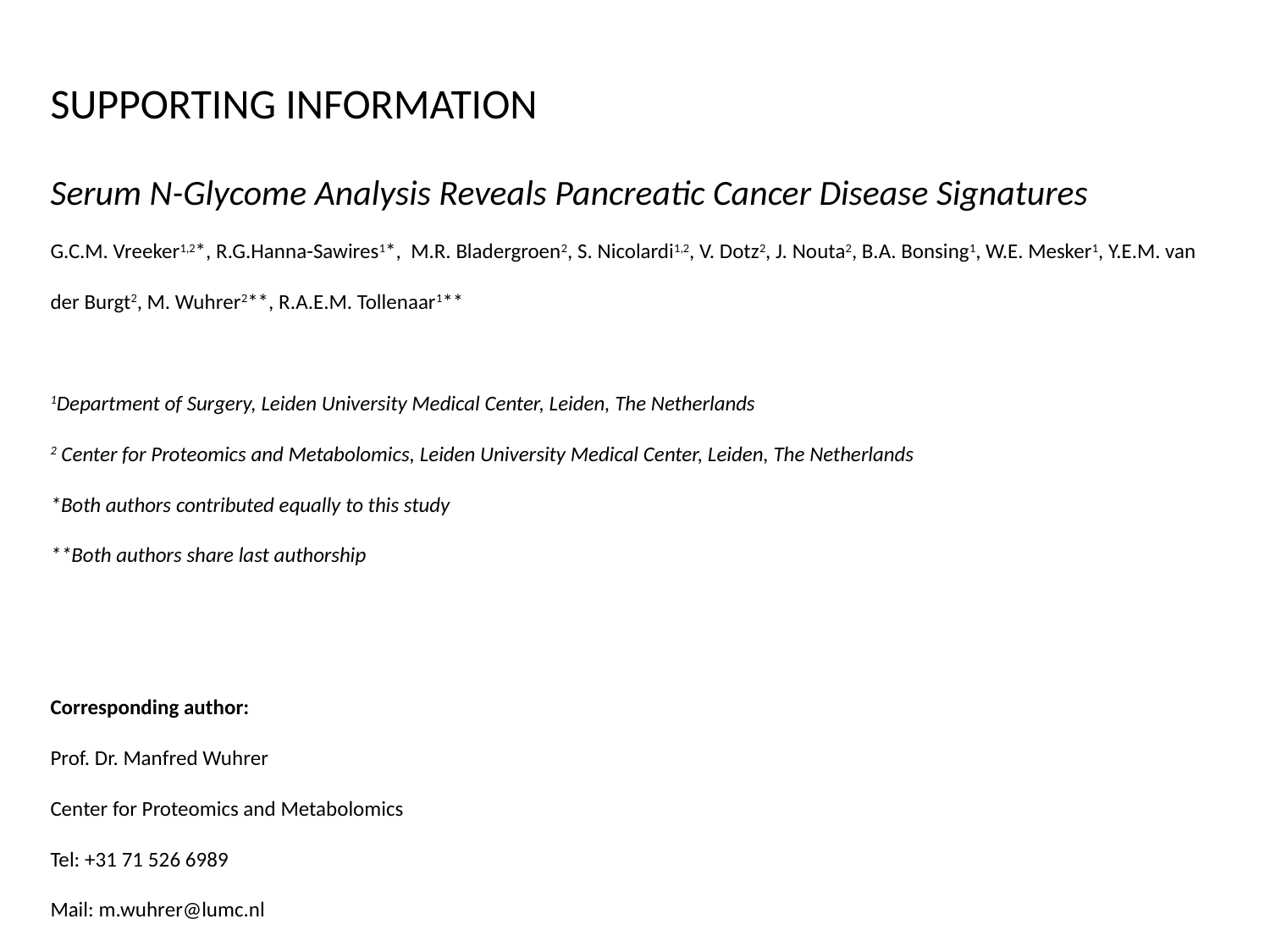

# SUPPORTING INFORMATION Serum N-Glycome Analysis Reveals Pancreatic Cancer Disease Signatures G.C.M. Vreeker1,2*, R.G.Hanna-Sawires1*, M.R. Bladergroen2, S. Nicolardi1,2, V. Dotz2, J. Nouta2, B.A. Bonsing1, W.E. Mesker1, Y.E.M. van der Burgt2, M. Wuhrer2**, R.A.E.M. Tollenaar1** 1Department of Surgery, Leiden University Medical Center, Leiden, The Netherlands 2 Center for Proteomics and Metabolomics, Leiden University Medical Center, Leiden, The Netherlands *Both authors contributed equally to this study **Both authors share last authorship   Corresponding author: Prof. Dr. Manfred Wuhrer Center for Proteomics and Metabolomics Tel: +31 71 526 6989 Mail: m.wuhrer@lumc.nl

## Slide 2
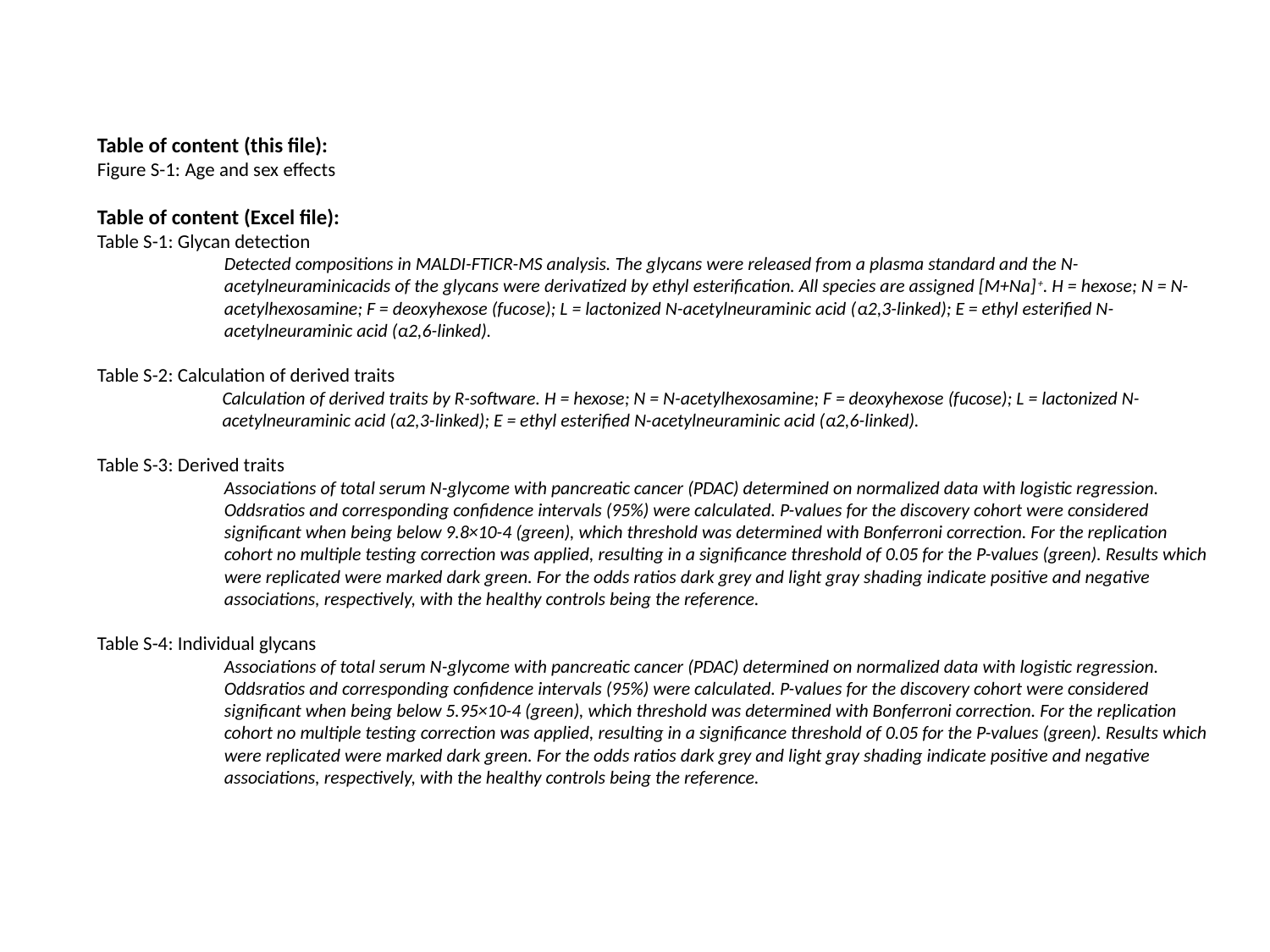

Table of content (this file):
Figure S-1: Age and sex effects
Table of content (Excel file):
Table S-1: Glycan detection
Detected compositions in MALDI-FTICR-MS analysis. The glycans were released from a plasma standard and the N-acetylneuraminicacids of the glycans were derivatized by ethyl esterification. All species are assigned [M+Na]+. H = hexose; N = N-acetylhexosamine; F = deoxyhexose (fucose); L = lactonized N-acetylneuraminic acid (α2,3-linked); E = ethyl esterified N-acetylneuraminic acid (α2,6-linked).
Table S-2: Calculation of derived traits
Calculation of derived traits by R-software. H = hexose; N = N-acetylhexosamine; F = deoxyhexose (fucose); L = lactonized N-acetylneuraminic acid (α2,3-linked); E = ethyl esterified N-acetylneuraminic acid (α2,6-linked).
Table S-3: Derived traits
Associations of total serum N-glycome with pancreatic cancer (PDAC) determined on normalized data with logistic regression. Oddsratios and corresponding confidence intervals (95%) were calculated. P-values for the discovery cohort were considered significant when being below 9.8×10^-4^ (green), which threshold was determined with Bonferroni correction. For the replication cohort no multiple testing correction was applied, resulting in a significance threshold of 0.05 for the P-values (green). Results which were replicated were marked dark green. For the odds ratios dark grey and light gray shading indicate positive and negative associations, respectively, with the healthy controls being the reference.
Table S-4: Individual glycans
Associations of total serum N-glycome with pancreatic cancer (PDAC) determined on normalized data with logistic regression. Oddsratios and corresponding confidence intervals (95%) were calculated. P-values for the discovery cohort were considered significant when being below 5.95×10^-4^ (green), which threshold was determined with Bonferroni correction. For the replication cohort no multiple testing correction was applied, resulting in a significance threshold of 0.05 for the P-values (green). Results which were replicated were marked dark green. For the odds ratios dark grey and light gray shading indicate positive and negative associations, respectively, with the healthy controls being the reference.

## Slide 3
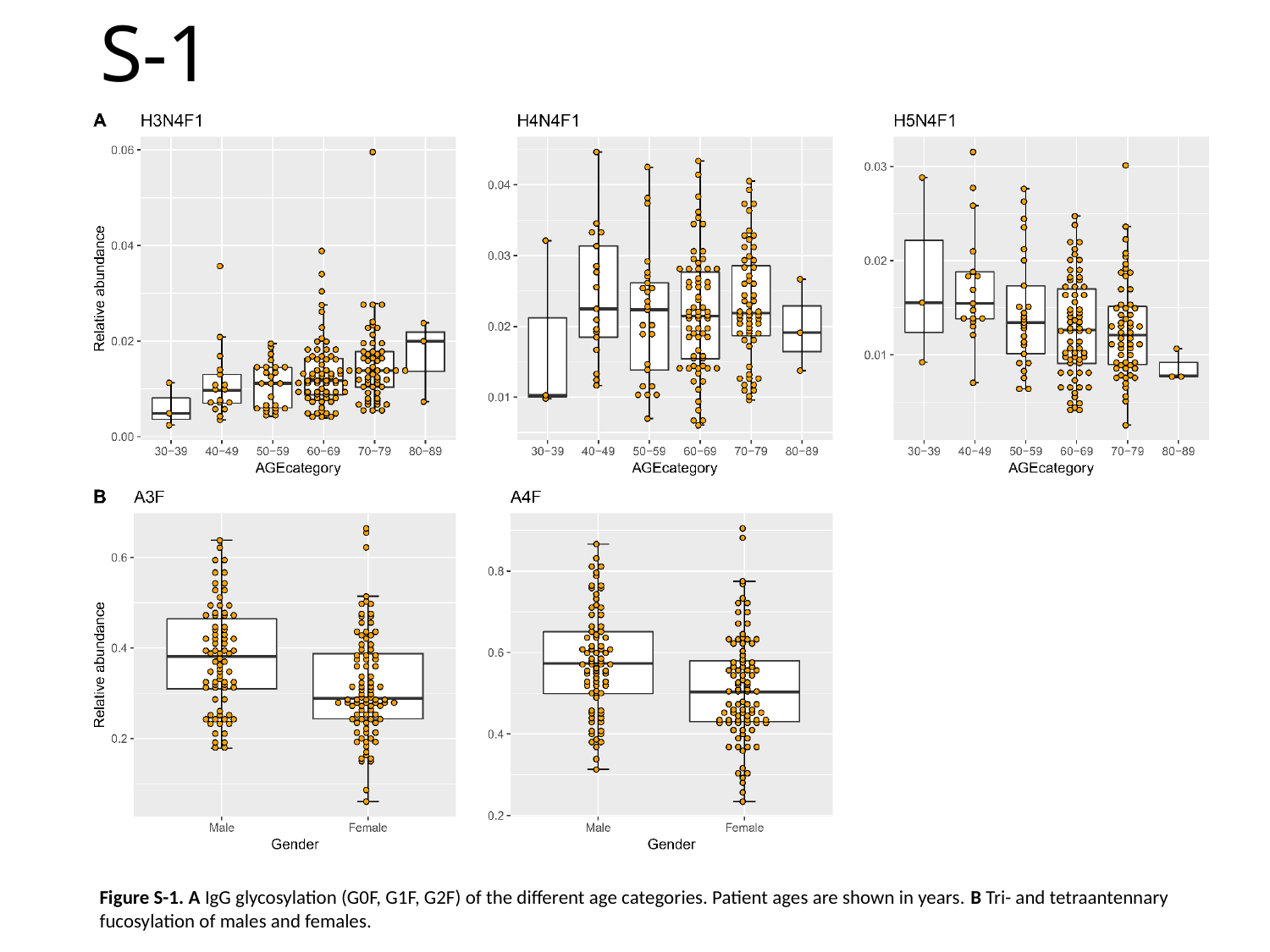

# S-1
Figure S-1. A IgG glycosylation (G0F, G1F, G2F) of the different age categories. Patient ages are shown in years. B Tri- and tetraantennary fucosylation of males and females.
